# *Arabidopsis* HISTONE DEACETYLASE 9 Stimulates Hypocotyl Cell Elongation by Repressing *GIGANTEA* Expression Under Short Day Photoperiod

**DOI:** 10.3389/fpls.2022.950378

**Published:** 2022-07-18

**Authors:** Hong Gil Lee, Yeong Yeop Jeong, Hongwoo Lee, Pil Joon Seo

**Affiliations:** ^1^Department of Chemistry, Seoul National University, Seoul, South Korea; ^2^Plant Genomics and Breeding Institute, Seoul National University, Seoul, South Korea; ^3^Research Institute of Basic Sciences, Seoul National University, Seoul, South Korea; ^4^Department of Biological Sciences, Sungkyunkwan University, Suwon, South Korea

**Keywords:** *Arabidopsis*, hypocotyl elongation, photoperiod, HDA9, *GI*

## Abstract

Developmental plasticity contributes to plant adaptation and fitness in a given condition. Hypocotyl elongation is under the tight control of complex genetic networks encompassing light, circadian, and photoperiod signaling. In this study, we demonstrate that HISTONE DEACETYLASE 9 (HDA9) mediates day length-dependent hypocotyl cell elongation. HDA9 binds to the *GIGANTEA* (*GI*) locus involved in photoperiodic hypocotyl elongation. The short day (SD)-accumulated HDA9 protein promotes histone H3 deacetylation at the *GI* locus during the dark period, promoting hypocotyl elongation. Consistently, *HDA9*-deficient mutants display reduced hypocotyl length, along with an increase in *GI* gene expression, only under SD conditions. Taken together, our study reveals the genetic basis of day length-dependent cell elongation in plants.

## Introduction

Hypocotyl elongation results from cell elongation, which is affected by various internal and environmental signals, such as phytohormone signaling, light quality and intensity, and ambient temperature (Gray et al., 1998; Niwa et al., 2009; Franklin et al., 2014; Gommers and Monte, 2018; Simon et al., 2018), and has been extensively investigated as a model for exploring the genetic networks controlling plant growth and development. PHYTOCHROME-INTERACTING FACTORS (PIFs), a group of basic helix-loop-helix (bHLH) transcription factors, are well-known as central players in hypocotyl elongation (Lorrain et al., 2008; Liu et al., 2011; Nusinow et al., 2011). Multilayered regulation of PIF4 and PIF5 at transcriptional and post-transcriptional levels underlies the elaborate control of hypocotyl growth (Kunihiro et al., 2010; Nusinow et al., 2011; Nieto et al., 2015; Hwang et al., 2019; Tavridou et al., 2020). Circadian clock components, such as PSEUDO-RESPONSE REGULATORS (PRRs) (Soy et al., 2016; Martin et al., 2018; Li et al., 2020), *GIGANTEA* (*GI*) (Li et al., 2016; Nohales et al., 2019), and the Evening Complex (Nusinow et al., 2011; Nieto et al., 2015), regulate *PIF4* and *PIF5* expression during the night (Nozue et al., 2007; Nusinow et al., 2011). Moreover, light-dependent degradation of PIFs by phytochromes further allows the accumulation of PIFs during the night (Nozue et al., 2007; Van Buskirk et al., 2014; Li et al., 2016; Pham et al., 2018; Hoang et al., 2021). The hormone- and temperature-dependent regulation of hypocotyl elongation also involves a PIF-dependent genetic pathway (Gray et al., 1998; Bernardo-Garcia et al., 2014; Li et al., 2016; Kim et al., 2020; Lee et al., 2021).

Notably, plants have evolved to coordinate their growth and development with seasonal variation in photoperiod (Wilczek et al., 2010). In *Arabidopsis*, diurnal hypocotyl elongation is accelerated to a greater extent in short days (SDs) than in long days (LDs), and requires both the circadian clock and light signaling to properly respond to the changing photoperiod (Nozue et al., 2007; Niwa et al., 2009). Several genetic factors are known to be involved in photoperiodic hypocotyl elongation. For example, the CYCLING DOF FACTOR (CDF) transcription factors, CDF1 and CDF5, which exhibit differential accumulation patterns under LD and SD conditions (Song et al., 2015), are crucial for hypocotyl elongation, especially in SDs (Martin et al., 2018, 2020). Consistently, genetic mutations of *CDF1* or *CDF5* reduce hypocotyl elongation in SDs but not in LDs (Martin et al., 2020), whereas constitutive overexpression of *CDF*s promotes hypocotyl growth only in SDs (Martin et al., 2020). In addition, *GI*, a key photoperiodic regulator, is also known to participate in hypocotyl elongation under various light conditions as shown by long hypocotyls under both red and blue light conditions (Huq et al., 2000; Tseng et al., 2004; Nohales and Kay, 2019; Park et al., 2020) and has been implicated in photoperiodic hypocotyl elongation (Kim et al., 2012; Li et al., 2016).

HISTONE DEACETYLASE 9 (HDA9) is involved in diverse aspects of plant growth and development, including seed dormancy (van Zanten et al., 2014; Baek et al., 2020), flowering time regulation (Kim et al., 2013, 2016; van Zanten et al., 2014; Kang et al., 2015; Mayer et al., 2019; Park et al., 2019; van der Woude et al., 2019; Zheng et al., 2020), leaf senescence (Chen et al., 2016; Mayer et al., 2019; Yang et al., 2020), cell differentiation (Lee et al., 2016), cell proliferation (Suzuki et al., 2018), stem cuticular wax accumulation (Wang et al., 2018), flower opening (Kang et al., 2015), and circadian oscillation (Lee et al., 2019). Moreover, HDA9 mediates responses to various environmental signals, such as salt and drought stresses (Zheng et al., 2016, 2020; Baek et al., 2020; Khan et al., 2020) and warm temperatures (Tasset et al., 2018; Shen et al., 2019; van der Woude et al., 2019). HDA9 negatively controls gene expression, in concert with interacting proteins, such as POWERDRESS (PWR), HIGH EXPRESSION OF OSMOTICALLY RESPONSIVE GENES 15 (HOS15), WRKY53, ELONGATED HYPOCOTYL 5 (HY5), ABA INSENSITIVE 4 (ABI4), and EARLY FLOWERING 3 (ELF3) (Chen et al., 2016; Lee et al., 2019; Mayer et al., 2019; Park et al., 2019; Baek et al., 2020; Khan et al., 2020).

In this study, we found that the HDA9–GI module is important for photoperiodic hypocotyl elongation. The HDA9 protein accumulated particularly in SDs, whereas *GI* was expressed to higher levels in LDs than in SDs. Under SD conditions, HDA9 could bind to the *GI* promoter and suppress its expression by removing histone H3 acetylation. Consistently, *hda9* and *gi* mutants displayed short and long hypocotyls, respectively, specifically under SD conditions. Overall, our results indicate that the negative regulation of *GI* by HDA9 is critical for hypocotyl elongation in SDs.

## Materials and Methods

### Plant Materials and Growth Conditions

*Arabidopsis thaliana* ecotype Columbia (Col-0) was used in this study. Seeds were plated on half-strength Murashige and Skoog (1/2 MS) medium containing 1% sucrose, and then cold-stratified for 2 days at 4 °C in continuous darkness. The plates were incubated at 22–23°C under long day (LD; 16 h light/8 h dark) or short day (SD; 8 h light/16 h dark) photoperiods and cool white fluorescent light (120 mmol photons m^–2^ s^–1^), to promote seed germination and seedling growth. The *hda9-1* (Suzuki et al., 2018; van der Woude et al., 2019), *hda9-2* (Suzuki et al., 2018; van der Woude et al., 2019), *gi-2* (Sawa and Kay, 2011), and *pHDA9:HDA9-VENUS/hda9-1* (Suzuki et al., 2018) plants have been described previously. Seeds of the T-DNA insertion mutants *hda9-1* (SALK_007123) and *hda9-2* (CS370750) were also obtained from the Arabidopsis Biological Resource Center (ABRC), Columbus, OH, United States. To measure hypocotyl length, seedlings were grown horizontally on a plate for 7 days, and hypocotyls were measured using the ImageJ software^1^. This experiment was conducted in at least three independent biological replicates, with each replicate containing at least 30 seedlings per genotype.

### Immunoblot Analysis

Total proteins were extracted from *pHDA9:HDA9-VENUS/hda9-1* seedlings grown for 7 days under LD and SD conditions. Whole seedlings were harvested and ground in liquid nitrogen. For the nuclear protein enrichment, the ground tissue was resuspended in nuclei isolation buffer [50 mM Tris–Cl (pH 7.5), 1 mM EDTA, 75 mM NaCl, 0.1% Triton X-100, 5% glycerol, 1 mM phenylmethylsulfonyl fluoride (PMSF), 1 × protease inhibitor cocktail]. After centrifugation at 15,000 × *g* for 10 min at 4 °C, the supernatant was discarded, and the pellet containing the nuclei was resuspended in 2 × sodium dodecyl sulfate (SDS) loading buffer. Nuclei suspension samples were transferred into clean tubes and 30 μg of each sample was separated by 10% SDS-polyacrylamide gel electrophoresis (PAGE). The HDA9-VENUS and histone H3 (loading control) proteins were immunodetected using rabbit anti-GFP antibody (1:5000 dilution; ab290; Abcam, Cambridge) and rabbit anti-histone H3 antibody (ab1791; Abcam, Cambridge), respectively.

### Quantitative Real-Time Reverse Transcription PCR

Total RNA was extracted using TransZol reagent (TransGen Biotech, Beijing, China) according to the manufacturer’s recommendations. Then, 2 μg of total RNA was reverse transcribed using Moloney Murine Leukemia Virus (M-MLV) reverse transcriptase (Dr. Protein, Seoul, South Korea) and dT18 oligos to synthesize first-strand cDNA. The cDNA samples were diluted to 100 μL with water, and 1 μL of diluted cDNA was used for PCR amplification.

Real-Time Reverse Transcription PCR reactions were performed in 96-well blocks on the Step-One Plus Real-Time PCR System (Applied Biosystems) using SYBR Master Mix (Enzynomics, Seoul, South Korea) and sequence-specific primers (Supplementary Table 5). The expression of genes was normalized relative to that of *EUKARYOTIC TRANSLATION INITIATION FACTOR 4A1* (*eIF4A*; At3g13920), and quantified using the ΔΔCt method. The specificity of the RT-qPCR primers was determined by melt curve analysis of the amplified products using the standard method installed in the system.

### Chromatin Immunoprecipitation Assays

Seven-day-old *pHDA9:HDA9-VENUS/hda9-1* and *hda9-1* seedlings grown under SD conditions were used for ChIP assays. The harvested plant materials were fixed in 1% formaldehyde and ground in liquid nitrogen. Chromatin was resuspended in nuclei lysis buffer [50 mM Tris–HCl (pH 8.0), 10 mM EDTA, 1% SDS, 1 mM PMSF, and 1× protease inhibitor cocktail] and sonicated using Bioruptor Pico (Diagenode) to generate approximately 500 bp fragments. The immunoprecipitates were collected using anti-GFP antibody (ab290; Abcam, Cambridge), anti-H3ac antibody (06-599; Millipore, Billerica, MA, United States), and agarose A/G beads (SC-2003; Santa Cruz Biotechnology, Santa Cruz, CA, United States), and DNA fragments were purified using the DNA elution kit (CMA0112, LaboPass, South Korea). The amount of precipitated DNA was quantified by quantitative PCR (qPCR) using sequence-specific primers (Supplementary Table 6). Data were normalized relative to *eIF4a*, and values for control plants were set to 1.

### Sequencing Data Analysis

The data of RNA-sequencing and ChIP-sequencing used in this study were obtained from previous studies. Up-regulated genes in *hda9* were analyzed using the data source of SRA BioProject number PRJNA743930. RNA-seq reads were mapped using STAR(v.2.7.10a) with ‘–pOverlapNbasesMin 12 –peOverlapMMp 0.1 –twopassMode Basic’ options (Dobin et al., 2013). RSEM(v1.3.1) was used to quantify transcript abundance (Li and Dewey, 2011), and DEseq2(v1.34.0) was used to identify differentially expressed genes (DEGs) between two conditions (Love et al., 2014) with cut-off of log_2_-fold change > 1 and *P*-value < 0.05. HDA9-binding genes were analyzed using the data source of accession number GSE80056 (Chen et al., 2016). ChIP-seq reads were trimmed and mapped using Trim Galore(v0.6.7)^2^ and Bowtie2(v2.4.5) (Langmead and Salzberg, 2012) with default parameters. MACS2(v2.2.7.1) (Zhang et al., 2008) was employed to call peaks with default parameters. Genes containing peaks within upstream 2-kb and gene body regions were annotated as HDA9-binding genes using ChIPpeakAnno(v3.28.1) (Zhu et al., 2010). Additionally, the list of down-regulated genes in response to darkness was obtained from Yan et al. (2019).

### Transient Expression Analysis Using *Arabidopsis* Protoplasts

For transient expression analysis using *Arabidopsis* protoplasts, coding sequences for *HDA9* and *GI* were cloned into the vector containing the CaMV 35S promoter. Seven-day-old seedlings grown under the SD conditions were harvested in 20 mL 0.5 M mannitol solution (90Φ plate) and incubated for 1 h at room temperature (RT) for protoplast extraction. Then, the 0.5 M mannitol solution was replaced to a 20 mL enzyme solution (2% Viscozyme L, 1% Celluclast 1.5 L, 1% Pectinex Ultra SP-L in MMC, adjusted to pH 5.8 by NaOH and sterilized through a 0.2 μm syringe filtering) and incubated in the darkness for 16 h at 22–23°C. The protoplasts were collected by centrifugation at 100 *g* for 7 min and washed twice with the W5 solution containing 0.1% glucose, 0.08% KCl, 0.9% NaCl, 1.84% CaCl2, and 2 mM MES (pH 5.7). *Arabidpsis* protoplasts were transfected through PEG-mediated transfection. After 16 h incubation in the dark at 23 °C, transformed protoplasts were processed for total RNA extraction.

## Results

### *hda9* Mutants Display Short Hypocotyls in Short Days

Accumulating evidence has shown that reversible histone acetylation and deacetylation underlie a variety of plant developmental processes. Here, we investigated hypocotyl elongation, which is regulated by environmental stimuli, such as ambient temperature, light intensity, and day length (Gray et al., 1998; Niwa et al., 2009; Franklin et al., 2014; Gommers and Monte, 2018). Notably, hypocotyl elongation is promoted under SD conditions (Nozue et al., 2007; Niwa et al., 2009), although the molecular basis of photoperiodic regulation of hypocotyl elongation remains to be fully elucidated.

**FIGURE 1:**
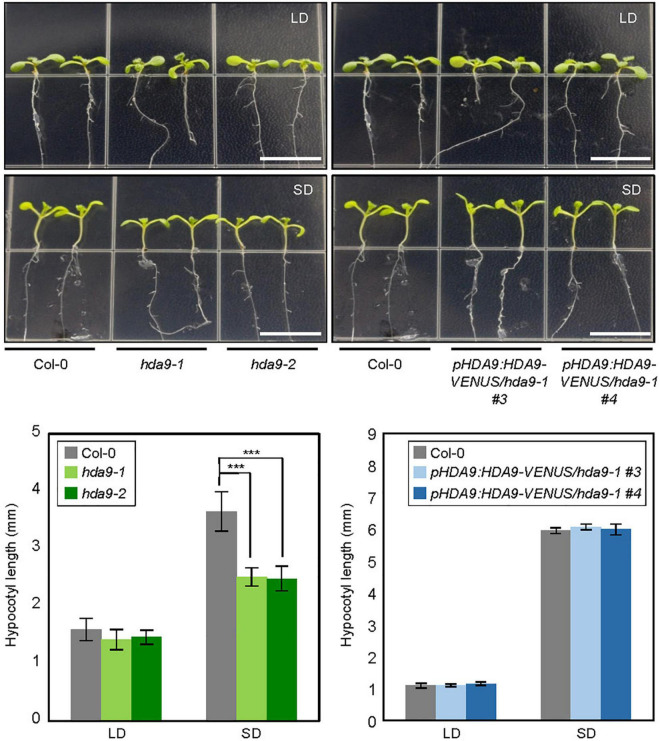
Hypocotyl elongation phenotypes of *hda9* mutants under short day (SD) photoperiod. Seedlings were grown under long day (LD) and SD conditions at 22°C, and hypocotyl length was measured for 7 days. At least 30 seedlings of each genotype were used in a single replicate, and values of three biologically independent replicates were averaged. Error bars indicate the standard error of mean (SEM). Statistical significance of the measurements was analyzed by Student’s *t*-test (***P < 0.001). Scale bars = 1.0 cm.

Initial screening revealed that the *HDA9* gene is particularly important for photoperiodic regulation of hypocotyl growth. We examined hypocotyl elongation in *HDA9*-deficient mutants, *hda9-1* and *hda9-2*, under both SD and LD conditions. Hypocotyl growth was reduced in both *hda9* mutants compared with the wild type in SDs; however, the hypocotyl length of both *hda9* mutants was indistinguishable from that of the wild type in LDs (Figure 1). To confirm the results, we tested the effect of photoperiod on hypocotyl elongation in *HDA9* complementation lines generated previously (Suzuki et al., 2018). The reduced hypocotyl length of *hda9-1* mutant in SDs was recovered in *pHDA9:HDA9-VENUS/hda9-1* seedlings (Figure 1). These results suggest that HDA9 promotes hypocotyl elongation under SD conditions.

### HDA9 Accumulation Is Enhanced in Short Days

**FIGURE 2:**
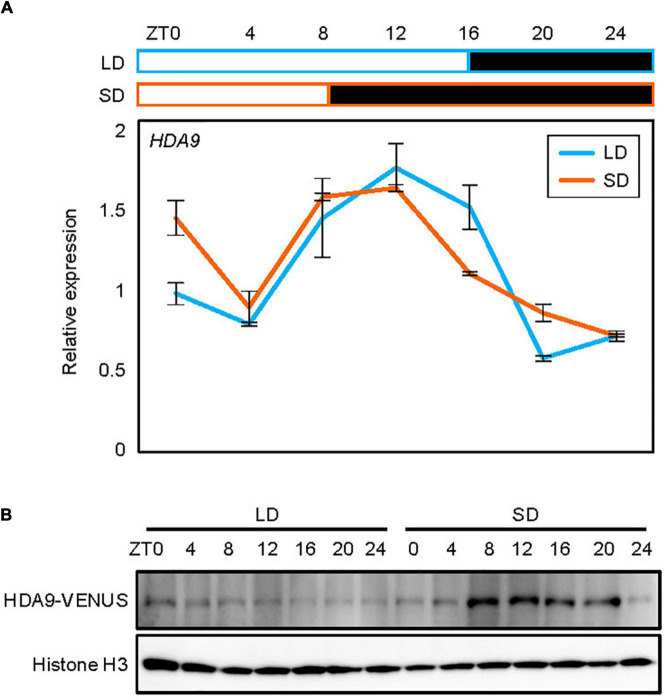
Accumulation of HDA9 protein in short days (SDs) relative to that in long days (LDs). Seedlings grown under LD and SD conditions for 7 days were harvested at 4 h intervals within a 24 h period. **(A)** Expression analysis of *HDA9*. Transcript levels of *HDA9* were analyzed by quantitative real-time reverse transcription PCR (RT-qPCR) and normalized relative to that of *eIF4A*. Error bars indicate the standard error of mean (SEM). White and black boxes indicate subjective day and night, respectively. **(B)** Analysis of nuclear HDA9 protein level. The HDA9-VENUS fusion protein in *pHDA9:HDA9-VENUS/hda9-1* plants were immunologically detected by Western blot analysis. Histone H3 accumulation was used as a loading control.

Since HDA9 promotes hypocotyl elongation specifically in SDs, we asked whether the *HDA9* gene is activated only under the SD photoperiod. Total RNA was isolated from 7-day-old seedlings grown in LDs and SDs, and subjected to RT-qPCR. The results showed that the diurnal expression pattern of *HDA9* was indistinguishable between seedlings grown under LD and SD conditions (Figure 2A and Supplementary Figure 1).

To further examine whether HDA9 accumulation is regulated at the translational and/or post-translational levels, we obtained transgenic plants expressing the *HDA9-VENUS* fusion under the control of the native *HDA9* promoter, and analyzed the diurnal pattern of HDA9 accumulation in LDs and SDs. Notably, while total HDA9 levels were unchanged between LDs and SDs (Supplementary Figure 2), the nuclear HDA9 protein accumulated to higher levels in SDs than in LDs, suggesting that day length-dependent nuclear localization and/or enrichment might be involved in photoperiodic hypocotyl elongation. The higher protein accumulation in SDs was particularly observed during the subjective night (ZT8–20) (Figure 2B). Although HDA9 accumulation increased during the dark period in SDs, it is unlikely that HDA9 accumulation was simply responsive to the dark, as the level of HDA9 protein remained unchanged during the dark period under LD conditions (Figure 2B). These observations suggest that the HDA9 protein level is mainly regulated by photoperiod rather than by dark exposure, and that SD-driven HDA9 accumulation is responsible for hypocotyl elongation.

### HDA9 Represses *GI* Expression in Short Days

**FIGURE 3:**
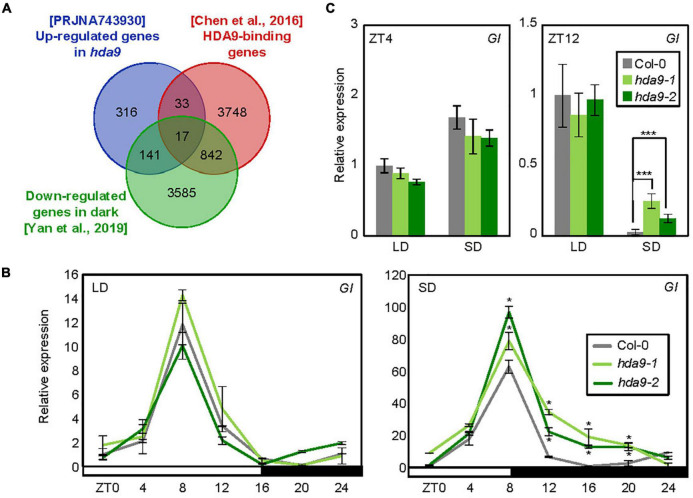
HDA9 represses *GIGANTEA* (*GI*) expression during the dark period in short days (SDs). **(A)** Venn diagram analysis. The intersection of genes targeted by HDA9, up-regulated in *hda9* mutants and down-regulated by darkness, was determined for downstream analysis. **(B)** *GI* expression in *hda9-1* and *hda9-2* mutants. Seven-day-old seedlings grown under long day (LD) and short day (SD) conditions (*n* > 15) were harvested at different time points within a 24 h period (from ZT0 to ZT24). Gene expression levels were normalized relative to *eIF4A* expression. Error bars indicate standard error of mean (SEM). **(C)** Transcript level of *GI* in *hda9* mutants in long days (LDs) and SDs. Whole seedlings were harvested at ZT4 and ZT12. Biological triplicates were averaged. Error bars indicate the SEM. Statistical significance of the measurements was analyzed by Student’s *t*-test (*P < 0.05).

Next, we investigated a molecular factor regulated by HDA9 to control photoperiodic hypocotyl elongation. We first obtained a list of HDA9 target genes from the HDA9-Flag ChIP-seq data published previously (Chen et al., 2016; Supplementary Table 1). To further narrow down the gene candidates responsible for HDA9-mediated regulation of photoperiodic hypocotyl elongation, we also collected genes repressed by HDA9 from Sequence Read Archive (SRA) data PRJNA743930 (Supplementary Table 2) as well as by darkness (Yan et al., 2019; Supplementary Table 3). Venn diagram analysis^3^ revealed 17 common genes (Figure 3A and Supplementary Table 4), including *GI*, *CDF3*, *INDOLE-3-ACETIC ACID INDUCIBLE 19* (*IAA19*), and *SMALL AUXIN UPREGULATED RNA 6* (*SAUR6*) (Supplementary Table 4).

We were particularly interested in investigating the *GI* gene, which is known as a key regulator of photoperiodic response (Kim et al., 2012; Song et al., 2014; Lee et al., 2019; Park et al., 2020) and has been shown to regulate hypocotyl elongation under various light conditions (Huq et al., 2000; Crepy et al., 2007; Li et al., 2016). We thus examined whether HDA9 regulates *GI* expression, and whether the effect of HDA9 on *GI* expression varies with day length. In wild-type seedlings, the expression of *GI* was diurnally regulated, with peak transcript levels at ZT8 in both LDs and SDs (Figure 3B). Notably, while the relative expression of *GI* was unaffected by absence of *HDA9* in LDs, its transcript level was increased in *hda9* mutants specifically under SD conditions, especially during the dark period (ZT8–20) (Figures 3B,C), when HDA9 accumulation was relatively high (Figure 2B). These results indicate that SD-induced accumulation of HDA9 represses *GI* expression, especially in dark period.

### HDA9 Directly Binds to the *GI* Promoter to Catalyze H3 Deacetylation in Short Days

**FIGURE 4:**
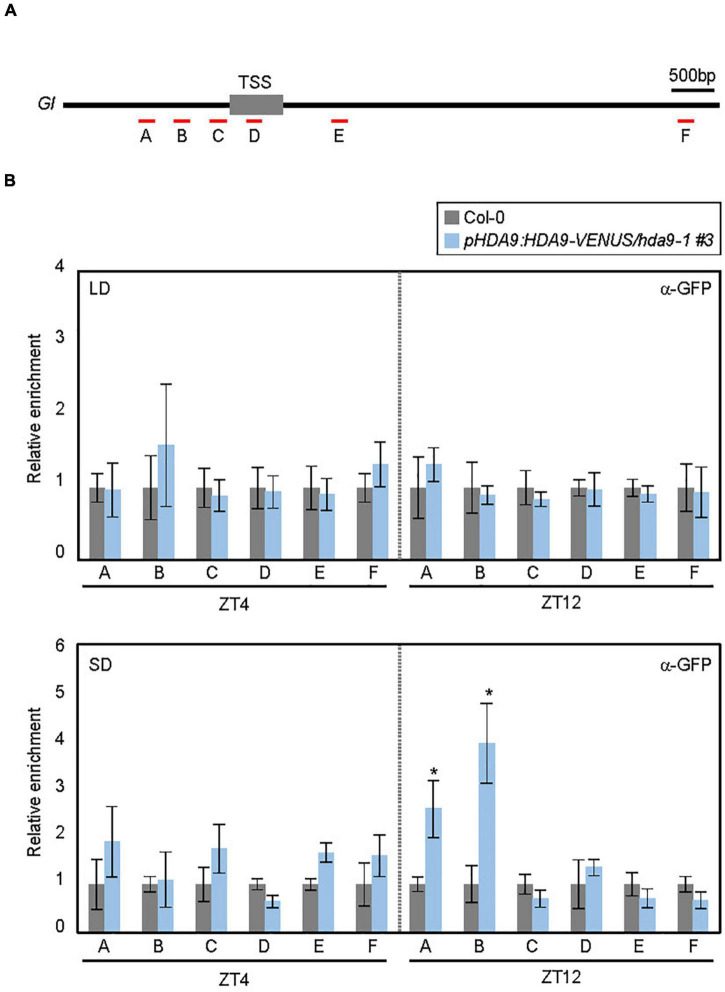
HDA9 binds to the *GIGANTEA* (*GI*) promoter. **(A)** Genomic structure of *GI*. Underbars indicate the regions amplified by PCR following chromatin immunoprecipitation (ChIP). **(B)** HDA9 binds to the *GI* locus in short days (SDs). Seven-day-old seedlings grown under long day (LD) and short day (SD) conditions were harvested at ZT4 and ZT12, and subjected to the ChIP assay using anti-GFP antibody. Three biological replicates were averaged. Error bars indicate the SEM. Statistical significance of the differences between Col-0 (wild type) and *pHDA9:HDA9-VENUS/hda9-1* transgenic plants were analyzed by Student’s *t*-test (*P < 0.05).

To investigate whether HDA9 directly binds to the *GI* locus, we performed ChIP assays using *pHDA9:HDA9-VENUS/hda9-1* transgenic plants grown under LD and SD conditions. ChIP-qPCR analysis of the plants harvested at ZT4 and ZT12 revealed that although HDA9 did not bind to the *GI* locus in LDs, its binding to the *GI* promoter was observed in SDs, specifically at ZT12 (Figures 4A,B). This is in agreement with the accumulation pattern of HDA9 (Figure 2B). Furthermore, the HDA9 protein showed strong association with the proximal region of the *GI* promoter, while other chromatin regions were not targeted by HDA9 (Figures 4A,B).

**FIGURE 5:**
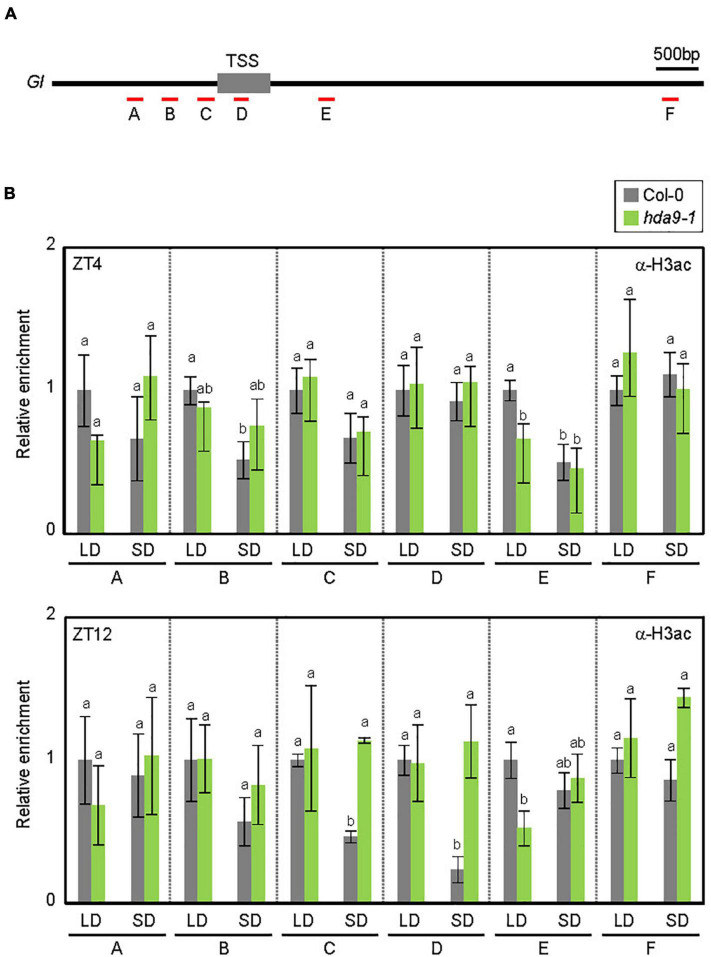
HDA9 regulates histone H3 acetylation levels at the *GIGANTEA* (*GI*) locus. **(A)** Genomic structure of *GI*. Underbars indicate the regions amplified by PCR following chromatin immunoprecipitation (ChIP). **(B)** HDA9 mediates the histone deacetylation at the *GI* locus in SDs. Seven-day-old seedlings grown under long day (LD) and short day (SD)conditions were harvested at ZT4 and ZT12, and subjected to the ChIP using anti-H3ac antibody. Three biological replicates were averaged. Error bars indicate the standard error of mean (SEM). Different letters represent a significant difference at *P* < 0.05 (one-way ANOVA with Fisher’s post hoc test).

The SD-specific recruitment of HDA9 to the *GI* locus was likely to facilitate photoperiodic histone deacetylation. Since HDA9 mainly determines histone H3 acetylation (H3ac) levels (Chen et al., 2016; Kim et al., 2016; Lee et al., 2019; Mayer et al., 2019; Baek et al., 2020), we examined H3ac accumulation at the *GI* promoter (Figures 5A,B). In wild-type seedlings, the H3ac level at the *GI* locus was higher in LDs, especially at ZT12, than in SDs (‘C’ and ‘D’ regions; Figure 5B), which is consistent with the higher expression level of *GI* in LDs (Figure 3C). The reduction of H3ac levels at the *GI* locus in SDs was likely caused by HDA9. Low H3ac deposition at ZT12 in SDs was at least partially impaired in the *hda9-1* mutant (Figure 5B). Consistent with the enhanced accumulation of HDA9 during the dark period in SDs (Figure 2B), the level of H3ac at the *GI* locus was unaffected in the *hda9-1* mutant at ZT4 under SD conditions (Figure 5B). Overall, our results indicate that HDA9 binds to and catalyzes histone deacetylation at the *GI* promoter to repress gene expression in SDs.

### HDA9–GI Module Ensures Photoperiodic Regulation of Hypocotyl Elongation

**FIGURE 6:**
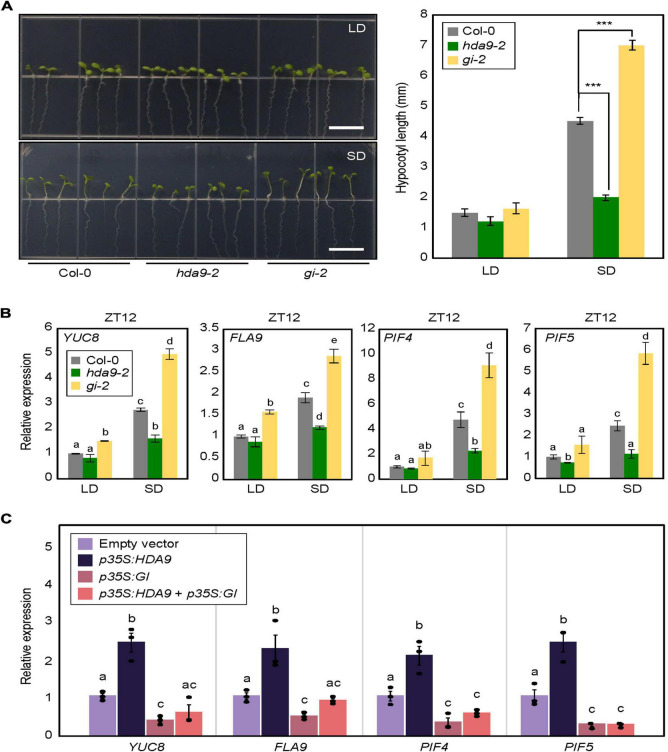
Photoperiodic regulation of hypocotyl elongation in *hda9-2* and *gi-2* mutants. **(A)** Analysis of hypocotyl growth in *hda9-2* and *gi-2* mutants in short days (SDs). Seven-day-old seedlings grown under long day (LD) and short day (SD) conditions were photographed (left panel). Hypocotyl length was measured using the ImageJ software (right panel). At least 30 seedlings of each genotype were used to measure the hypocotyl length. Three biological replicates were averaged. Error bars indicate the standard error of mean (SEM). Statistical significance of the measurements was analyzed by Student’s *t*-test (***P < 0.001). Scale bars = 1.0 cm. **(B)** Expression analysis of *YUC8, FLA9, PIF4*, and *PIF5* at ZT12. Transcript accumulation was measured by RT-qPCR. Three biological replicates were averaged. Error bars indicate the SEM. Different letters represent a significant difference at *P* < 0.05 (one-way ANOVA with Fisher’s *post hoc* test). **(C)** Expression of *YUC8*, *FLA9, PIF4*, and *PIF5* in protoplasts transiently overexpressing *HDA9* and *GIGANTEA* (*GI*). Seven-day-old seedlings grown under SD conditions were used for protoplast isolation. The recombinant constructs were coexpressed transiently in *Arabidopsis* protoplasts, and then protoplast samples were harvested at ZT12 24 h after transfection to extract total RNAs. Transcript accumulation was determined by RT-qPCR analysis. Biological triplicates were averaged, and statistical significance of the measurements was analyzed by one-way analyzed by one-way analysis of variance (ANOVA) with Fisher’s *post hoc* test (*P* < 0.05). Bars indicate the standard error of the mean.

Our results showed that HDA9 directly binds to the *GI* promoter and represses its expression, particularly under SD conditions. Although the role of *GI* in hypocotyl elongation has been demonstrated under various light conditions (Tseng et al., 2004; Kim et al., 2012; Li et al., 2016), its role in photoperiodic hypocotyl elongation needed to be verified under our conditions. Therefore, we measured the hypocotyl length of the *GI*-deficient mutant (*gi-2*) plants in LDs and SDs. Under the LD photoperiod, the *gi-2* mutant displayed marginally increased hypocotyl length compared with wild-type seedlings (Figure 6A). However, in SDs, the *gi-2* mutant exhibited much longer hypocotyls than the wild type (Figure 6A), whereas the *hda9-2* mutant displayed shorter hypocotyl length (Figure 6A), indicating that the HDA9–GI module plays a critical role in photoperiodic regulation of hypocotyl elongation.

To further support these observations, we examined the expression of hypocotyl elongation-related genes, including *YUCCA 8* (*YUC8*), *FASCICLIN-LIKE ARABINOOGALACTAN 9* (*FLA9*), *PIF4*, and *PIF5*, in *gi-2* and *hda9-2* mutants. In LDs, all of these genes showed comparable expression levels in wild-type and mutant seedlings. However, under SD conditions, these genes were significantly downregulated in *hda9-2* seedlings but upregulated in *gi-2* seedlings (Figure 6B), in agreement with their hypocotyl phenotypes (Figure 6A).

We wanted to confirm genetic hierarchy between *HDA9* and *GI*. To this end, 35S:*HDA9* and 35S:*GI* constructs were generated and used for transient expression analysis using protoplasts obtained from SD-grown seedlings. Overexpression of *HDA9* led to the increase in *YUC8*, *FLA9, PIF4*, and *PIF5* expression, whereas *GI* overexpression lowered their expression in transfected protoplasts (Figure 6C). Notably, overexpression of both *HDA9* and *GI* reduced the expression of all genes comparable to *GI* overexpression (Figure 6C), indicating that *GI* is epistatic to *HDA9* in the control of photoperiodic hypocotyl elongation.

**FIGURE 7:**
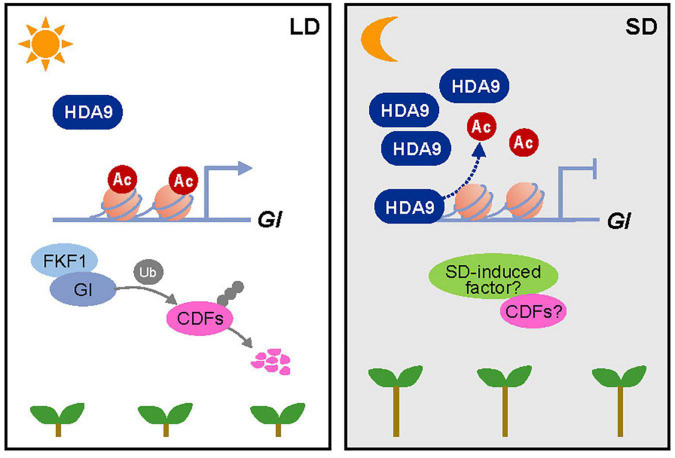
Proposed working model of the HDA9–GI module demonstrating its role in photoperiodic hypocotyl elongation. In short days (SDs), the HDA9 protein accumulates at dusk and then binds to the *GIGANTEA* (*GI*) promoter. This binding of HDA9 to the *GI* promoter facilitates the removal of H3ac from the region surrounding the transcription start site (TSS) of *GI*, thus repressing its expression in SDs. The HDA9–GI module regulates hypocotyl elongation in a photoperiod-dependent manner, possibly by acting together with as-yet-unidentified SD-activated molecular factors.

Taken together, our results show that HDA9 accumulates by SD conditions and directly binds to the *GI* locus to repress its expression. When *GI* reaches peak expression in SDs, HDA9 promotes H3 deacetylation at the *GI* promoter in the dark to reduce its expression during the night, thereby promoting hypocotyl elongation (Figure 7). Given the SD-specific impact of mutations in the *GI* gene on hypocotyl elongation, we speculate that *GI* and/or its downstream target are involved in crosstalk with SD-activated factors (see Discussion).

## Discussion

Plants have evolved sophisticated molecular mechanisms for sensing changes in day length and inducing appropriate physiological responses. The representative signaling axis has been demonstrated in the control of photoperiodic flowering. Under LD conditions, GI interacts with the F-box protein FLAVIN-BINDING, KELCH REPEAT, F-BOX 1 (FKF1) in a blue-light-dependent manner, inducing the 26S proteasome-dependent degradation of CYCLING DOF FACTOR 1 (CDF1), which acts as a floral repressor by directly repressing the expression of *CONSTANS* (*CO*) (Imaizumi et al., 2005; Sawa et al., 2007). The FKF1–GI complex-induced *CO* transcription promotes expression of the *FT* gene (Imaizumi et al., 2003, 2005; Sawa et al., 2007; Song et al., 2012). By contrast, under the SD photoperiod, FKF1 and GI do not interact, and their inability to form the degradation complex leads to low *CO* transcript abundance (Sawa et al., 2007; Song et al., 2014). In parallel, *GI* transcriptionally activates the transcription of *miR172*, which inhibits expression of *TARGET OF EAT1* (*TOE1*), a negative regulator of *FT* in LDs (Jung et al., 2007). Additionally, GI binds directly to the *FT* promoter, possibly to activate its expression in LDs (Sawa and Kay, 2011). Furthermore, GI also binds to the transcriptional repressors of *FT*, such as SHORT VEGETATIVE PHASE (SVP), TEMPRANILLO 1 (TEM1), and TEMPRANILLO 2 (TEM2) (Sawa and Kay, 2011). The degradation of *FT* transcriptional repressors or unavailability of corresponding binding sites in the *FT* promoter because of the presence of *GI* relieves *FT* from transcriptional repression (Sawa and Kay, 2011; Jang et al., 2015). Consistently, *gi* mutants are non-responsive to day length, and display constitutive late flowering under both LD and SD conditions (Redei, 1962; Mizoguchi et al., 2005).

Hypocotyl elongation is promoted particularly in SDs (Niwa et al., 2009). The photoperiodic regulation of hypocotyl elongation most likely involves *GI*-regulated signaling components. The SD-repressed *GI* accumulation allows photoperiod-dependent hypocotyl growth, probably *via* multiple routes. *GI* promotes the degradation of CDFs in LDs (Fornara et al., 2009); however, post-translational regulation of CDFs by *GI* may be impaired in SDs. CDF proteins accumulated under SD conditions act as positive regulators of hypocotyl elongation (Martin et al., 2018, 2020). Consistently, the *cdf5* mutant shows shorter hypocotyls only in SDs, whereas constitutive overexpression of *CDF5* promotes hypocotyl growth under SD conditions (Martin et al., 2020). The *gi* mutant and *CDF5*-overexpressing seedlings showed no difference in hypocotyl length, compared with wild type, under LD conditions (Martin et al., 2020; Park et al., 2020), suggesting that *GI*- and CDF-regulated hypocotyl elongation requires additional SD-activated components. For instance, CDFs may interact with SD-induced factors to robustly promote hypocotyl elongation in SDs (Figure 7).

*GI* may also affect *PIF4* expression. It has been demonstrated that while *PIF4* expression levels are unaffected in *gi* mutants under LD conditions (Kim et al., 2012), its expression is promoted in *gi* mutants under SD conditions (Kim et al., 2012). PIF activity is gated during the dark phase in SDs and induces the expression of growth-promoting genes, including auxin signaling and other hormone-related genes (Michael et al., 2008; Nozue et al., 2011; Li et al., 2016; Soy et al., 2016; Martin et al., 2018). *CDF5* is also regulated by PIFs. While *CDF5* expression is maintained at low levels from the subjective day to early night by sequential waves of PRRs, PIFs activate *CDF5* expression during the subjective night, inducing cell elongation (Martin et al., 2018, 2020). Moreover, it is also possible that GI acts as a positive regulator of TOC1 or in parallel with TOC1 to regulate hypocotyl elongation (Martin-Tryon et al., 2007).

Although GI is known as a pivotal regulator of photoperiodic responses in plants, how the activity of GI is shaped by the photoperiod remains elusive. In this study, we found that SD-activated HDA9 represses *GI* expression, and the HDA9–GI module regulates hypocotyl elongation only in SDs. HDA9 binds to the *GI* locus and catalyzes H3 deacetylation in SDs. Importantly, the regulation of *GI* by HDA9 was relevant particularly in SDs. The activity of HDA9 is also likely associated with skotomorphogenesis, since the *hda9* mutants displayed reduced hypocotyl elongation in darkness (Supplementary Figure 3). Consistently, the accumulation of HDA9 protein was also increased in response to the continuous dark treatment (Supplementary Figure 4). However, the role of HDA9 in skotomorphogenesis is likely independent of *GI* because the *gi-2* mutant showed no defect in skotomorphogenesis (Supplementary Figure 3; Huq et al., 2000; Tseng et al., 2004). Overall, our results demonstrate that HDA9 regulates *GI* expression in a photoperiod-dependent manner, ultimately allowing photoperiodic plant development.

## Data Availability Statement

Publicly available datasets were analyzed in this study. The original data were deposited in the NCBI repository, accession numbers: PRJNA743930 and GSE80056.

## Author Contributions

PJS and HGL participated in the design of the study and wrote the manuscript. HGL and YYJ executed the molecular experiments. HL performed the bioinformatic analysis of sequencing data. All authors approved the submitted version.

## Conflict of Interest

The authors declare that the research was conducted in the absence of any commercial or financial relationships that could be construed as a potential conflict of interest.

## Publisher’s Note

All claims expressed in this article are solely those of the authors and do not necessarily represent those of their affiliated organizations, or those of the publisher, the editors and the reviewers. Any product that may be evaluated in this article, or claim that may be made by its manufacturer, is not guaranteed or endorsed by the publisher.

## Footnotes

1
http://rsb.info.nih.gov/ij

2
https://www.bioinformatics.babraham.ac.uk/projects/trim_galore/

3
http://bioinformatics.psb.ugent.be/webtools/Venn/
