# Supplementary material for: Arabidopsis HISTONE DEACETYLASE 9 Stimulates Hypocotyl Cell Elongation by Repressing GIGANTEA Expression Under Short Day Photoperiod
Source: Front Plant Sci. 2022 Jul 18;13:950378. doi: 10.3389/fpls.2022.950378 (PMC9341324; doi:10.3389/fpls.2022.950378)
Supplement: Supplementary file 1 [file Data_Sheet_1.pdf]

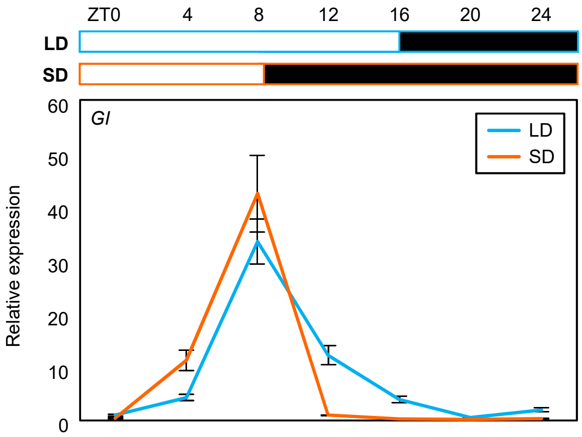

Supplementary Figure 1. Diurnal expression of *GI* under LD and SD conditions. Seven-day-old seedlings grown under LD and SD conditions were harvested from ZT0 to ZT24 to analyze transcript accumulation of *GI*. Gene expression values were normalized relative to *eIF4A* expression. Bars indicate the standard error of the mean. The white and black boxes indicate the subjective day and night, respectively.

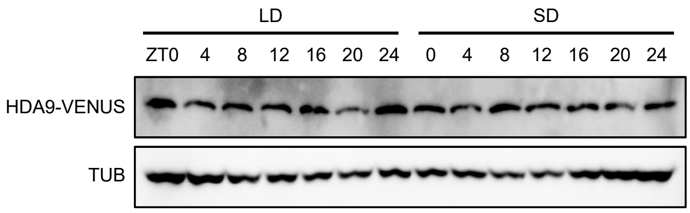

Supplementary Figure 2. Total protein accumulation of HDA9. Seven-day-old seedlings grown under LD and SD conditions were harvested from ZT0 to ZT24 to analyze protein accumulation of HDA9. The α-TUB protein was immunologically detected using mouse anti-α-Tubulin antibody (T9026, Sigma, USA) as a loading control.

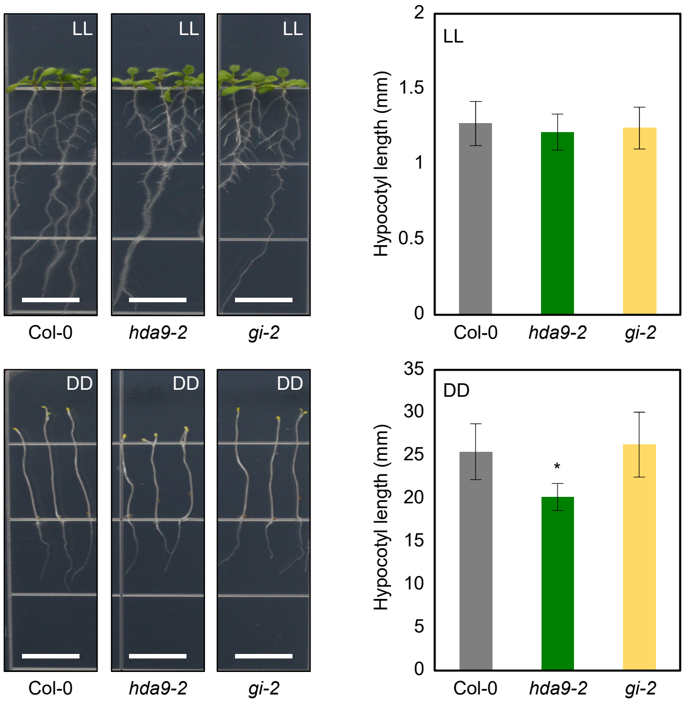

Supplementary Figure 3. Hypocotyl lengths in *hda9-2* and *gi-2* mutants under LL and DD conditions. Seedlings germinated for 2 days in LL were transferred to LL and DD conditions for 5 days. At least 30 seedlings were averaged to measure hypocotyl length. Bars indicate the standard deviation of the mean. Statistically significant difference was determined by Student's *t*-test (*$P < 0.05$). Scale bars = 1.0 cm.

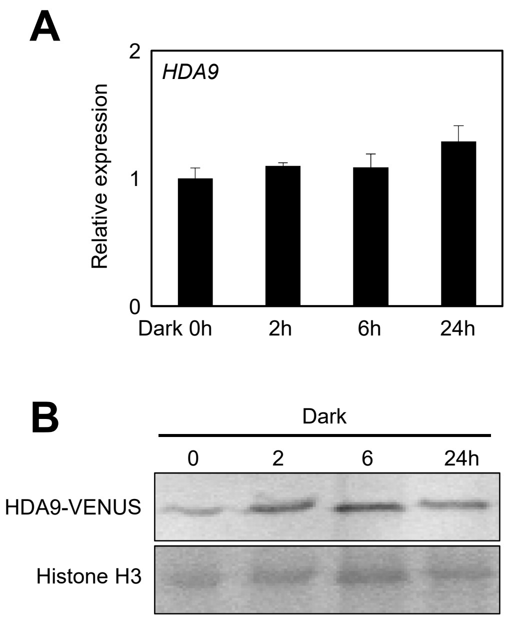

Supplementary Figure 4. Transcript and protein levels of HDA9 in darkness. Seven-day-old seedlings grown under continuous light conditions (LL) were transferred to darkness. Whole seedlings (n > 15) were harvested to analyze transcript and protein accumulation. **(A)** Transcript accumulation of *HDA9*. Gene expression values were normalized relative to *eIF4A* expression. Biological triplicates were averaged. Bars indicate the standard error of the mean. **(B)** Protein accumulation of HDA9. The histone H3 protein was detected with rabbit anti-histone H3 antibody (ab1791, Abcam, Cambridge) as a loading control.

Supplementary Table 5. List of RT-qPCR primers used in this study.
The sizes of PCR products ranged from 80 to 300 nucleotides in length. F, forward primer; R, reverse primer.

Primer	Usage	Sequence
eIF4a-F	RT-qPCR	5′-TGACCACACAGTCTCTGCAA
eIF4a-R	RT-qPCR	5′-ACCAGGGAGACTTGTTGGAC
HDA9-F	RT-qPCR	5′-GCCTGCATAGCAAGATGGAA
HDA9-R	RT-qPCR	5′-CCGGCGTAAAGTTGACAAAA
GI-F	RT-qPCR	5′-AGCAGCAGCAGTTGTCCTTC
GI-R	RT-qPCR	5′-TAGCACCGGCTGTATTGCTC
YUC8-F	RT-qPCR	5′-AGAAACTTCCATGCCGGAGT
YUC8-R	RT-qPCR	5′-GTTCTTGTCGTCGGATGTGG
FLA9-F	RT-qPCR	5′-CTCTCGCAGTTTATGTTGTCG
FLA9-R	RT-qPCR	5′-GGATTTAGATTTAGGGGCAGG
PIF4-R	RT-qPCR	5′-AGATCATCTCCGACCGGTTT
PIF4-R	RT-qPCR	5′-CGCCGGTGAACTAAATCTCA
PIF5-F	RT-qPCR	5′-GCTCCAAGCACAGAACCAAA
PIF5-R	RT-qPCR	5′-GCACGGTCTGCATCTGATTT

Supplementary Table 6. List of primers used in chromatin immunoprecipitation (ChIP) assays. F, forward primer; R, reverse primer.

Primer	Sequence
GI(A)-F	CCAAAGCCCAGTTATGTTACC
GI(A)-R	GCAATAAGATGTGAGCAATA
GI(B)-F	ATTTGTTGAGTGGGCTTGAAT
GI(B)-R	TCTCTCATGTGATTGCCTAAT
GI(C)-F	TACTTAAATTGGCAACATTTTTCTTGGTG
GI(C)-R	CCACATACATCTCCACCCATACCATG
GI(D)-F	CCAACAAACTCATAGGAAGAC
GI(D)-R	TAGAAACAGTGATTTACCAAG
GI(E)-F	GATTGCAAACTAAAGATATGAAAG
GI(E)-R	CTCAGTTTTATAAATGGGACGGT
GI(F)-F	GCAACTGATGGAATGCTTG
GI(F)-R	TAGCACCGGCTGTATTGCTC
